# Supplementary material for: Pro-Inflammatory Chemokines CCL5, CXCL12, and CX3CL1 Bind to and Activate Platelet Integrin αIIbβ3 in an Allosteric Manner
Source: Cells. 2022 Sep 29;11(19):3059. doi: 10.3390/cells11193059 (PMC9563052; doi:10.3390/cells11193059)

Supplemental Figures

Figure S1. Effect of antagonists to αIIbβ3 on chemokine binding to αIIbβ3.
Well of 96-well microtiter plate were coated with chemokines (50 μg/ml coating concentrations) and remaining protein binding sites were blocked with BSA. Wells were incubated with soluble αIIbβ3 (1 μg/ml) in the presence of mab 7E3, eptifibatide in Tyrode-HEPES buffer with 1 mM Mn^{2+} for 1 h at room temperature ans bound αIIbβ3 was quantified using anti-human β3 mAb (AV10) and HRP-conjugated anti- mouse IgG. Data is shown as means +/- SD in triplicate experiments.

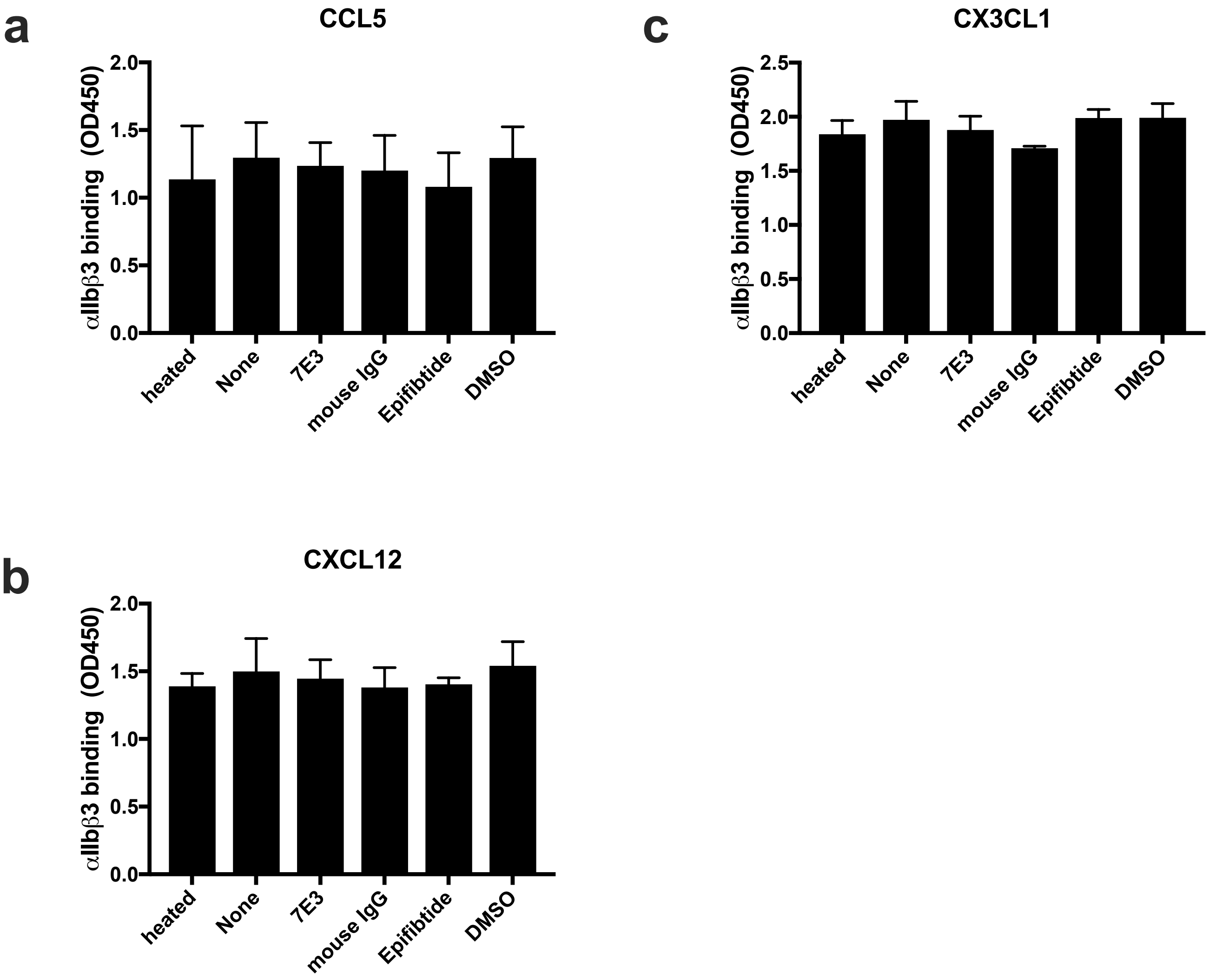

Figure S2. Chemokines enhanced binding of soluble αIIbβ3 to immobilized fibrinogen fragment γC390-411 fused to GST.

Well of 96-well microtiter plate were coated with fibrinogen fragment γC390-411 (20 μg/ml) and remaining protein binding sites were blocked with BSA. Wells were incubated with soluble αIIbβ3 (1 μg/ml) in the presence of chemokines at the indicated concentrations in Tyrode-HEPES buffer with 1 mM Ca^{2+} for 1 h at room temperature ans bound αIIbβ3 was quantified using anti-human β3 mAb (AV10) and HRP-conjugated anti- mouse IgG. Data is shown as means +/- SD in triplicate experiments.

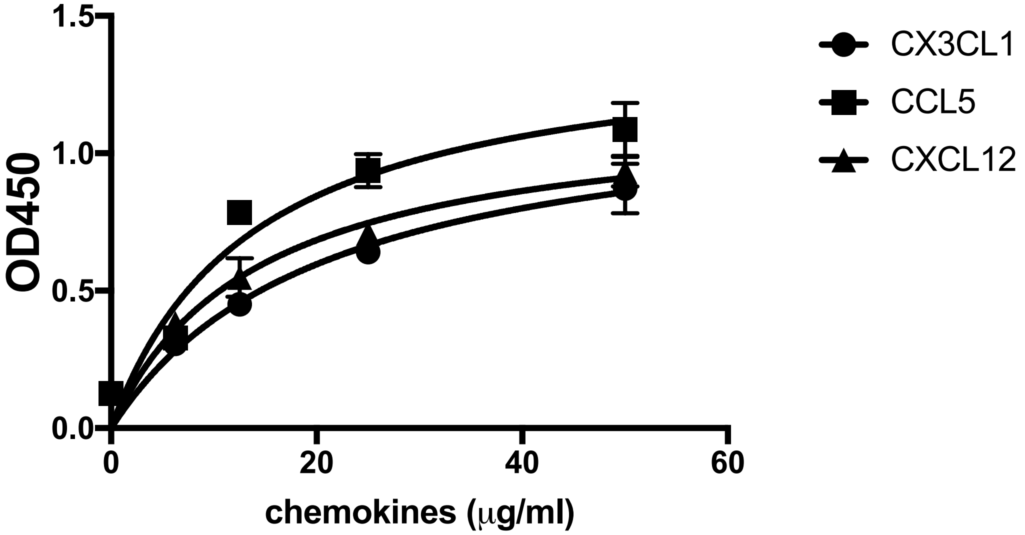

Figure S3. Chemokines enhanced binding of soluble αIIbβ3 to immobilized γC151-411 peptide fused to GST.

Well of 96-well microtiter plate were coated with fibrinogen fragment γC151-411 (50 μg/ml) and remaining protein binding sites were blocked with BSA. Wells were incubated with soluble αIIbβ3 (1 μg/ml) in the presence of chemokines at the indicated concentrations in Tyrode-HEPES buffer with 1 mM Ca^{2+} for 1 h at room temperature ans bound αIIbβ3 was quantified using anti-human β3 mAb (AV10) and HRP-conjugated anti- mouse IgG. Data is shown as means +/- SD in triplicate experiments.

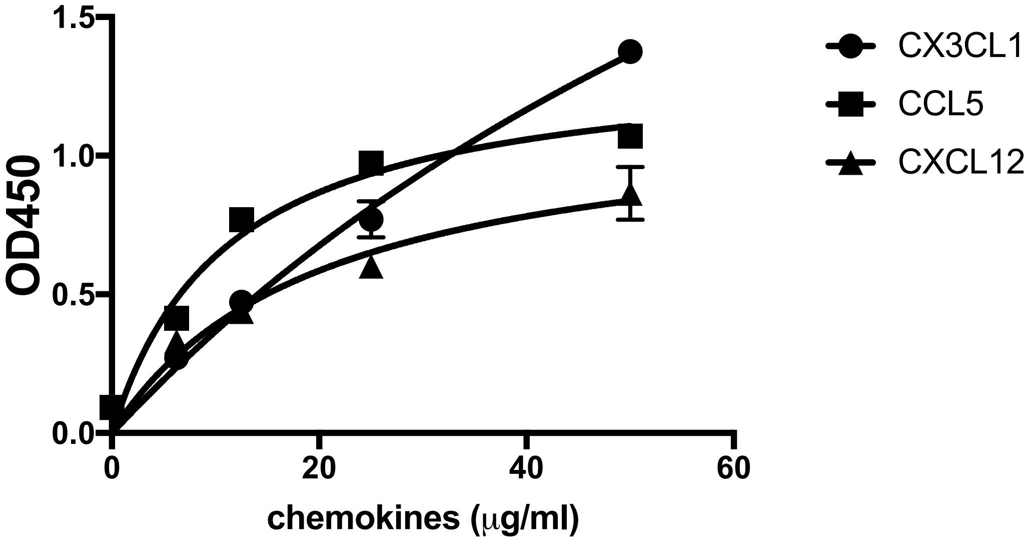

Figure S4. Activation of αIIbβ3 on CHO cells (αIIbβ3-CHO cells) by chemokines does not induce LIBS2 epitopes. Cells were incubated with chemokines (10 μg/ml) and anti-LIBS2 (10 μg/ml) in Tyrode-HEPES buffer with 1 mM Ca^{2+} (total 100 μl) for 30 min on ice. Bound IgG was detected with FITC-labeled anti-mouse IgG in flow cytometry. Data is shown as means +/- SD in triplicate experiments.

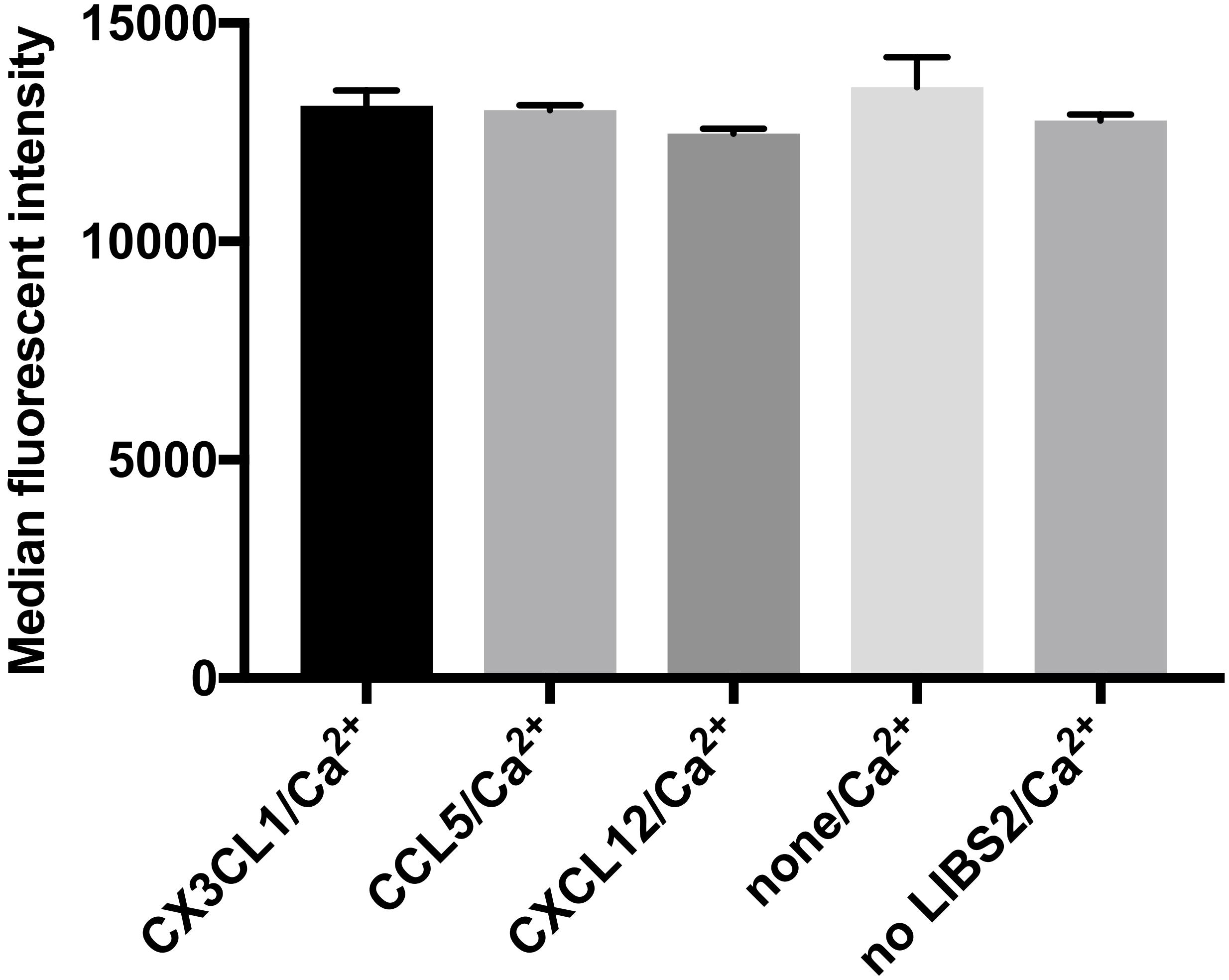

Supplement: Supplementary file 1 [file cells-11-03059-s001.zip › cells-1934234-supplementary.pdf]
